# Actissist: Proof-of-Concept Trial of a Theory-Driven Digital Intervention for Psychosis

**DOI:** 10.1093/schbul/sby032

**Published:** 2018-03-16

**Authors:** Sandra Bucci, Christine Barrowclough, John Ainsworth, Matthew Machin, Rohan Morris, Katherine Berry, Richard Emsley, Shon Lewis, Dawn Edge, Iain Buchan, Gillian Haddock

**Affiliations:** 1 Division of Psychology and Mental Health, School of Health Sciences, Manchester Academic Health Science Centre, University of Manchester, Manchester, UK; 2 Division of Informatics Imaging and Data Sciences, University of Manchester, Manchester, UK; 3 Health eResearch Centre, Farr Institute for Health Informatics Research, University of Manchester, Manchester, UK; 4 Division of Population Health, Health Services Research and Primary Care, University of Manchester, Manchester, UK; 5 Microsoft Research, Cambridge, UK

**Keywords:** psychosis, relapse, mHealth, digital intervention, randomized controlled trial, early psychosis

## Abstract

**Background:**

Timely access to intervention for psychosis is crucial yet problematic. As such, health care providers are forming digital strategies for addressing mental health challenges. A theory-driven digital intervention that monitors distressing experiences and provides real-time active management strategies could improve the speed and quality of recovery in psychosis, over and above conventional treatments. This study assesses the feasibility and acceptability of Actissist, a digital health intervention grounded in the cognitive model of psychosis that targets key early psychosis domains.

**Methods:**

A proof-of-concept, single, blind, randomized controlled trial of Actissist, compared to a symptom-monitoring control. Thirty-six early psychosis patients were randomized on a 2:1 ratio to each arm of the trial. Actissist was delivered via a smartphone app over 12-weeks; clinical and functional assessment time-points were baseline, post-treatment and 22-weeks. Assessors’ blind to treatment condition conducted the assessments. Acceptability was examined using qualitative methods.

**Results:**

Actissist was feasible (75% participants used Actissist at least once/day; uptake was high, 97% participants remained in the trial; high follow-up rates), acceptable (90% participants recommend Actissist), and safe (0 serious adverse events), with high levels of user satisfaction. Treatment effects were large on negative symptoms, general psychotic symptoms and mood. The addition of Actissist conferred benefit at post-treatment assessment over routine symptom-monitoring and treatment as usual.

**Conclusions:**

This is the first controlled proof-of-concept trial of a theory-driven digital health intervention for early psychosis. Actissist is feasible and acceptable to early psychosis patients, with a strong signal for treatment efficacy.

Trial Registration: ISRCTN: 34966555.

Psychosis onset typically occurs in early adulthood, a critical period for psychosocial development. Despite initial response to intervention, the early course of psychosis is characterized by repeated relapse,^1^ compromising functional and social development,^2^ service engagement,^2^ and the resilience of carers and services.^3^ Meta-analyses have shown that discontinuation of antipsychotic medication, substance misuse, family criticism, poorer premorbid functioning, and social isolation are firmly associated with relapse following a first episode of psychosis (FEP^1^). Early intervention for psychosis services (EIS) exist worldwide and aim to provide both pharmacological and psychosocial interventions. Despite mandates published to address the treatment gap,^4^ timely access to these services is problematic.^5^

Health care providers worldwide are forming digital strategies for addressing mental health challenges, and self-management in long-term conditions is now a cornerstone of many national health policies.^6^ Smartphones are commonplace technology that can deliver unconstrained, real-time packages of care, extending the reach of health care delivery. Smartphone-extended care could drive improvements in quality, efficiency, cost, and access to treatment, while enhancing patient experience by: providing more choices over *how* health care is delivered; facilitating self-management; and assisting clinicians to gain a richer understanding of an individual’s day-to-day experiences by receiving real-time data that can be used to deliver ecologically valid treatment. Smartphone ownership rates in psychosis are comparable to the general population^7^ and use of smartphones for health care appears acceptable to people with severe mental health problems.^8^ Large-scale meta-analyses of randomized controlled trials (RCTs) have demonstrated that digital health interventions (DHIs) can provide effective treatment for mental health problems such as depression^9^ and anxiety.^10^ While a number of smartphone-delivered open trials have shown promising effects in reducing hospital admissions, improving positive psychotic symptoms, socialization, social connectedness, depression, and medication adherence (see review^1^), a user-informed, theory-driven app tested in early psychosis has not been reported in the literature. As such, the lack of controlled trials in the field precludes firm conclusions regards feasibility, acceptability, and intervention effects in this group.^11^

We report here on Actissist, a DHI that is broadly designed to speed up recovery and improve quality of care. The Actissist system is unconstrained by traditional service settings and can be used for active self-management and facilitate shared-decision making about treatment. Grounded in the cognitive model of psychosis, and following an extensive period of co-design with patients and stakeholders, Actissist is more specifically a theory-informed smartphone app targeting key early psychosis domains. The system swiftly identifies and challenges unhelpful appraisals of psychosis-related experiences and provides alternative, more helpful coping strategies in the real-time context of one’s daily life. Actissist was informed by content described in various published academic works.^12–21^ We draw on experience sampling methodology to prompt participants to engage with the app and build on the clinical protocols described by Granholm et al^2^ and Ben-Zeev et al.^22^ Using an agile, iterative process of development, beta-testing and with user experience design (UX) in mind, Actissist targets 5 domains associated with early psychosis relapse: auditory verbal hallucinations; paranoia; perceived criticism; socialization; and cannabis use.

The overarching aim of this Medical Research Council (MRC)-funded trial was to establish proof-of-concept evidence that the Actissist intervention is feasible and acceptable in early psychosis compared with a symptom-monitoring app as an active control condition.^23^ In line with MRC guidelines for developing complex interventions,^24^ the a priori focus of the trial was estimation of treatment effects. The study had 2 aims: (1) test the safety, feasibility, and acceptability of the Actissist intervention; (2) provide preliminary evidence of intervention effects on clinical and functional outcomes. This is the first RCT of a DHI targeting putative mechanisms for early psychosis against an active control condition.

## Methods

### Study Design

A single blind, proof-of-concept, pilot RCT of 36 early psychosis patients with random allocation using a 2:1 ratio to receive either Actissist plus treatment as usual (TAU; *n* = 24) or ClinTouch (a symptom monitoring app) plus TAU (*n* = 12) over 12 weeks. Trained researchers blind to treatment allocation completed study assessments at time 1 (baseline), time 2 (12-weeks, post-treatment), and time 3 (22 weeks). Eligibility criteria were: (1) in current contact with an EIS in the North West of England; (2) capacity to provide informed consent; and (3) English language proficient. EIS are multidisciplinary community mental health services that provide psychosocial and pharmacological treatment and support to people in their first 3 years of their initial episode of psychosis. Exclusion criteria were: (1) aged less than 16 years at point of recruitment; (2) not capable of giving informed consent; (3) non-English proficient; and (4) inpatient at point of recruitment. Inclusion criteria were as broad as possible to improve the external validity of the trial. The trial was prospectively registered (ISRCTN34966555) and received ethical approval from the National Research Ethics Committee West Midlands—South Birmingham (14/WM/0118).

### Recruitment and Randomization

Participants were recruited over 7 months from several NHS Trusts in the North West of England. Health professionals working within EIS identified eligible participants and passed on the contact details of those who consented to be contacted to either a trained researcher or a clinical studies officer (CSO) from the UK Clinical Research Network who supported recruitment to the trial. Following consent to contact, the researcher or CSO invited and consented participants into the trial. Following baseline assessment, participants were randomized in a 2:1 random allocation designed to maximize information about the Actissist intervention. Since hypothesis testing was not the objective of this study, a sample size of 36 was chosen a priori to assess feasibility, conduct preliminary statistical analyses, and obtain parameters to inform a robust power calculation for a fully powered efficacy trial.

Where possible, randomization occurred within 2 working days of baseline assessment. Receipt of the Actissist or control app typically commenced within 2 working weeks of randomization. The study statistician produced a randomization list using random permuted blocks of size 3 and 6. Notification of group allocation occurred using an independent tool (eLabs^25^; NWEH^26^), an online research platform that concealed group allocation. The study coordinator was unblinded to treatment allocation and participants were informed about the outcome of randomization from a researcher after the baseline assessment. Many strategies were used to protect blinding, including researchers working on different days to minimize overlap, researchers not being involved in the randomization process, considering room use and diary arrangements, and reminding participants not to disclose group allocation. Group allocation was revealed only to the participant, responsible clinician, baseline research assessor, and project officer. Overall, there was only one blind break in the treatment group; another rater masked to group allocation completed each respective assessment when unblinding occurred. Accordingly, all ratings used for analysis were masked.

### Measures

The primary outcome was feasibility, which was assessed in terms of uptake (the proportion of eligible participants consenting to the study), attrition, the proportion of participants completing user, and alert-initiated data entries across participants (>33% data points), and the proportion continuing for 12 weeks (both arms). Acceptability of the Actissist intervention was assessed via participant feedback. Once written informed consent had been obtained, trained researchers administered a battery of secondary outcome measures (details of measures reported elsewhere^27^). Demographic information was collected as well as measures of frequency, intensity, and distress of psychotic symptoms (Positive and Negative Syndrome Scale, PANSS^28^; PSYRATS,^29^ depression (Calgary Depression Scale for Schizophrenia, CDSS^30^), functioning (Global Assessment of Functioning scale, GAF, APA, 1994^31^; Personal and Social Performance Scale, PSP^32^), empowerment (Empowerment Rating Scale, ERS^33^), health status and health-related quality of life (EQ-5D-5L^34^). Frequency and quantity of alcohol and cannabis use (Timeline Follow Back, TLFB^35^), perceived criticism from significant others (perceived criticism scale^36^), medication adherence and attitudes to medication (Medication Adherence Rating Scale, MARS^37^), and satisfaction with technology^38^ was also measured.

All measures were administered at each assessment time-point. Participants were reimbursed £20 for completing assessment time-points. Assessors underwent a rigorous training process and received weekly supervision by the chief investigator. Researchers also attended monthly PANSS supervision groups run by experienced senior clinical academics for the duration of the study period. All assessors met departmental reliability standards after pretrial training (mean ICC = 0.89 PANSS total score across the 4 raters), followed by regular supervision in administration, scoring procedures and inter-rater reliability checks over the course of the trial period.

### Procedure

Full details of the procedure are reported elsewhere.^27^ Briefly, participants in both conditions received a 45-min phone set-up training session focused on basic use of the smartphone (eg, charging the phone; on/off), demonstration of the app (both conditions), setting a passcode, and navigating participants through the app domains and settings. For Actissist, participants watched an in-built video explaining the basic principles of cognitive therapy, the theoretical orientation upon which the Actissist app is based. For ClinTouch, participants were provided a rationale for symptom monitoring. In both apps, participants could also view written and visual “in-app” instructions. Participants had the opportunity to use the app and ask questions. They were instructed to charge the phone regularly, to carry the phone at all times, and to go about their daily life as usual. No restrictions were placed on smartphone use.

Following smartphone demonstration, participants were instructed to use the app for 12 weeks. Participants were instructed to respond to alerts wherever possible and were encouraged to use the on-demand features as and when needed. All participants received a weekly phone call from the project manager to troubleshoot equipment functions. Software was either preloaded on a loaned smartphone with £10 and topped up remotely each month to support data connectivity over the trial period or at their request downloaded on to the participant’s own smartphone. Participants using their own handset were given £10/month to cover data usage costs. Engagement with the apps was incentivized; a £10 shopping voucher was given to participants on a fortnightly basis over the intervention period who completed at least one-third of data entry points. A criterion was applied to determine whether an entry contributed to the overall app engagement algorithm. Specifically, there was a maximum of 3 valid entries per day. To prevent participants from artificially inflating their app usage to achieve the app usage incentives, where a participant self-initiated use (or where there was a combination of self- and prompted- initiation) which exceeded 3 entries per day, only the first 3 entries contributed to the engagement figure. Serious adverse events (SAEs) were strictly monitored, reviewed and documented by the team and discussed with a nominated senior clinical academic and trialist independent to the team.

### Interventions

#### Actissist

**Fig. 1.**
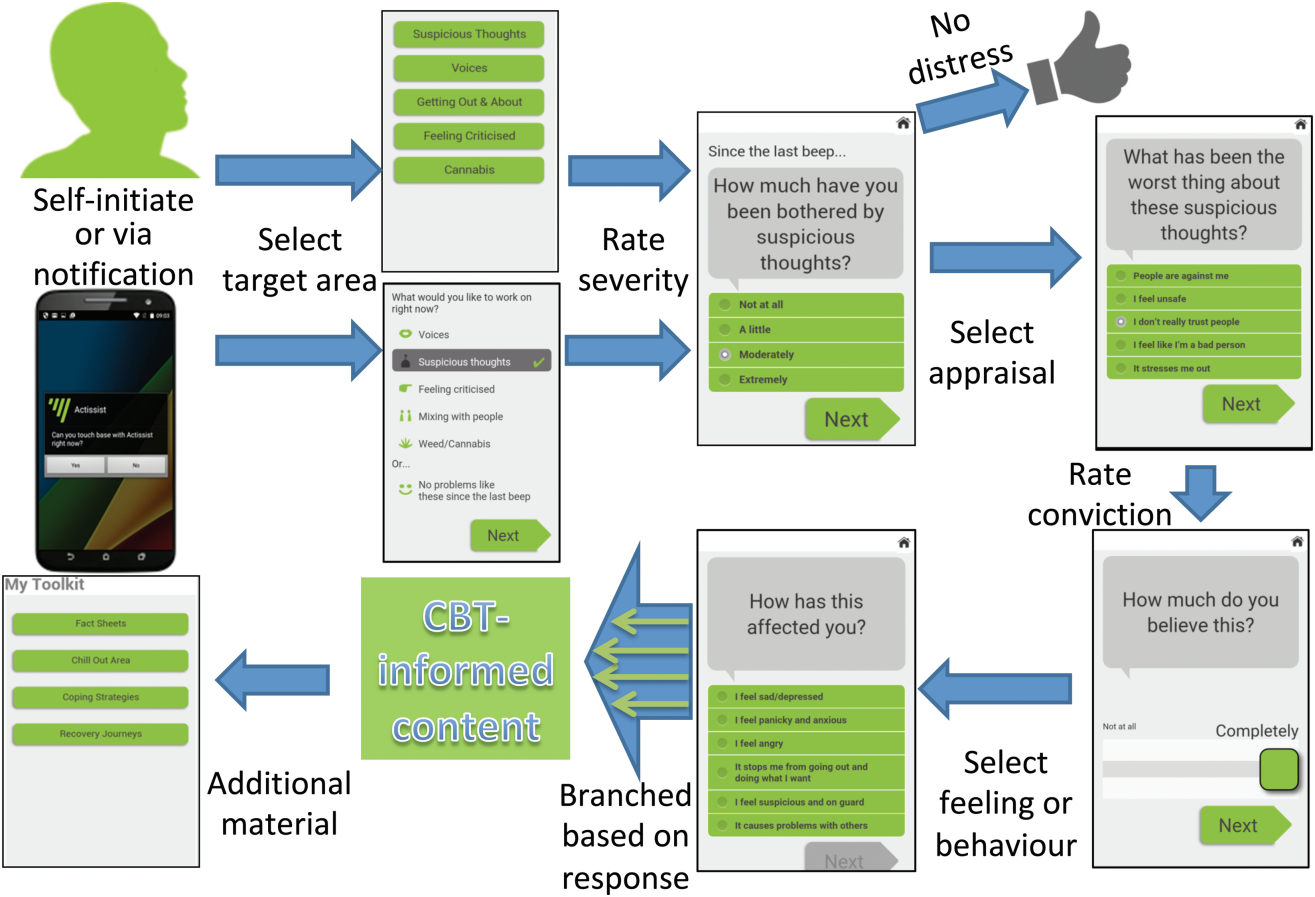
Schematic of Actissist intervention, including screen shots of the Actissist app.

Actissist is a DHI that the user can engage with spontaneously or in response to being prompted. It then collects responses from the user and wirelessly uploads user responses to a server. Actissist is divided in 2 parts, although presented as a single app. Firstly, at 3 pseudo-randomized time points per day, 6 days a week between 10.00 and 22.00, an auditory alert followed by a visual prompt is emitted from the app inviting participants to access the app. The notifications persist on the handset (ie, no time out) until such point as they are accepted, dismissed, “snoozed” (up to 15 min), or another notification is received. The notifications serve merely as a reminder; the app also allows self-initiated use at any point. If a user accepts a notification or initiates use they are invited to select an intervention domain(s) and then complete a series of self-assessment questions structured as question-answer exchanges that focus on cognitive appraisals, belief conviction, emotions and associated behaviours. Depending on the appraisal selected, the exchange is followed by normalizing messages and cognitive or behavioral strategies aimed at suggesting ways of coping with distressing experiences. Multiple messages and images associated with each exchange minimize boredom and repetition within the app. Alternatively, participants can report that they have had “no problems like this” since their last notification or (self-initiated) interaction. Part 2 includes a menu of multi-media options that act in a stand-alone fashion designed to complement and support the feedback from the intervention domains. This supplementary content contains information and activities including relaxation and mindfulness exercises, recovery stories (videos), a range of fact sheets (eg, low mood, anxiety, self-esteem), external links to web-related content (eg, TED talks), daily diary, and emergency contacts resources. Furthermore, a graphical summary of data points entered over the previous 7 days allows users to track distressing experiences to support active self-management of symptoms and shared decision making about treatment with clinicians. Users can customize the aesthetics of the Actissist interface; for example, personally meaningful images from the smartphone’ s local storage can be set as wallpaper to facilitate positive mood induction. Figure 1 displays a visual schematic of the system, including screenshots of the Actissist app.

#### ClinTouch (Control Condition)

The ClinTouch app is a symptom-monitoring app that triggers, collects, and wirelessly uploads symptom data to a server. As in the treatment condition, the app emits an alarm prompting participants to access the app at 3 pseudo-randomized time points per day, 6 days a week between 10.00 and 22.00 for 12 weeks alongside usual treatment. The ClinTouch protocol is outlined in detail in Palmier-Claus et al^23^; although, the number of prompts was altered for parity with Actissist alerts, such that participants submit one-and-a-half data points daily with 10 branching items covering positive psychotic symptoms, anxiety, and mood. As each full data point was collected over 2 separate alerts, this equates to having received 3 alerts every day. The alert invites participants to use a touchscreen slider to rate the severity of 12 individual symptoms on a 1–7 scale. This takes an average of 70 s and the data are wirelessly uploaded to a secure server. Symptom items have been validated against corresponding items on the PANSS.^28^ The aesthetics and interface mirror the Actissist interface. However, unlike the Actissist app, ClinTouch does not facilitate self-initiated access; data entries must be in response to a notification. The notifications time out 30 min after receipt whereby the notification is no longer visible and the ClinTouch items are no longer accessible.

To minimize risk, we did not store identifying data on either the app or the server. Participants set a passcode to access the smartphone. Three general principles of information security (confidentiality, integrity, and availability) were followed in the design and implementation of the trial. All data transmitted to and from the servers was encrypted over https with strong ciphers as detailed in the Approved Cryptographic Algorithms Good Practice Guidelines.^39^

TAU involved regular clinician meetings, medication, risk monitoring, and psychosocial interventions. Actissist and ClinTouch are standalone apps that do not link with services.

## Statistical Analysis

Analyses follow the CONSORT 2010 Statement,^40^ showing referral and attrition (ie, participant flow) and an a priori analysis plan was published.^27^ Analyses were undertaken in Stata (version 14.1) after completion of the endpoint assessment. The primary outcome (feasibility) was assessed in terms of uptake (the proportion of eligible participants consenting to join the study), attrition, proportion of participants completing user and alert-initiated data entries across participants, and the proportion continuing for 12 weeks (both arms). Demographic data were presented using descriptive statistics. Ecological momentary interventions typically operationalize >33% data points completed as evidence of compliance,^41,42^ which was the “accept” criterion for compliance that we adopted. The “target” criterion was 50% of participants submitting 50% of data entries. Linear regression was used to examine the effect of random allocation on the secondary outcomes at 12 and 22 weeks separately, adjusting for outcome measures at baseline. We report adjusted mean differences and their standard errors, Cohen’s *D* standardized effect sizes and their corresponding bootstrapped 95% confidence intervals (CIs), based on adjusted mean differences and the pooled standard deviation at baseline. Acceptability was assessed by participant feedback using a semi-structured interview. Feedback was thematically organized.

## Results

### Sample Characteristics and Feasibility Outcomes

**Fig. 2.**
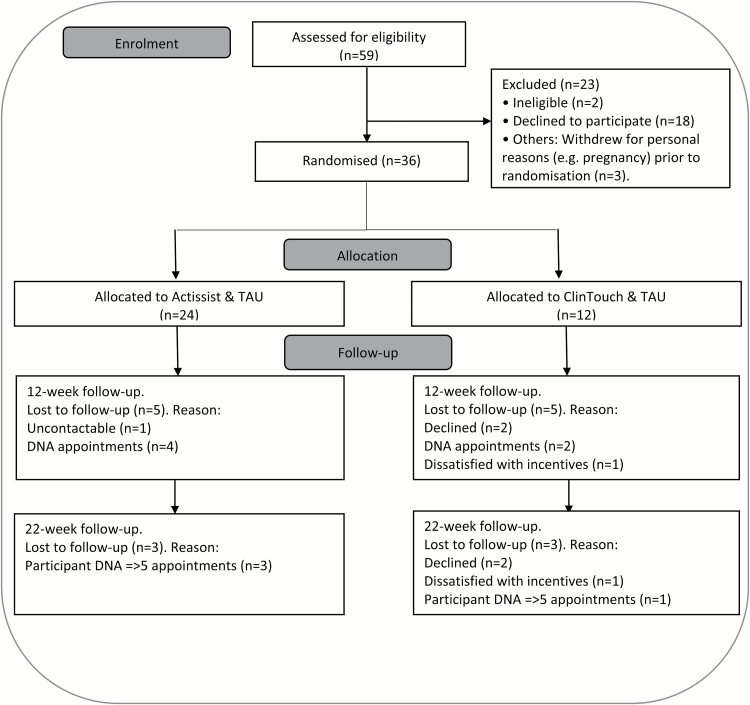
CONSORT diagram.

A summary of demographic and clinical information for all participants is displayed in tables 1 and 2. There were very few drug users in the sample (2 cannabis, 2 cocaine, 1 mephodrone, 1 gogaine); 14/36 participants smoked cigarettes regularly. As can be seen in figure 2, uptake into the trial was high: 38/59 people (64.4%) referred participated to the full trial. Reasons for declining participation included medication side effects, concentrating on studies/employment, and not wishing to focus on mental health at the current time. Retention in the Actissist arm and tolerance for the Actissist app was excellent, as evidenced by no participant withdrawals. The “accept” criterion for data points completed was met in both trial arms (75% and 50% participants, respectively, submitting >33% data entries as per our pre-specified criteria). In other words, 75% of Actissist participants used the app on average at least once a day over the 12-week intervention period, suggesting excellent engagement and acceptability of the Actissist app. The “target” criterion was achieved in the Actissist arm only (63% vs 42% in ClinTouch). All participants except one (97%) remained in the trial (both arms) until the end. The participant who withdrew from the study returned the phone and withdrew from EIS all together (nonresearch related incident). No research-related SAEs were recorded for any participants during the study period, suggesting that both apps are safe. Completion of assessments was also high: 72% (26/36) and 83% (30/36) participants successfully followed up at post-treatment and 22 weeks, respectively.

**Table 1.** Demographic Characteristics: Means (SD) or Numbers (%) of Participants

	Actissist (*n* = 24)	ClinTouch (*n* = 12)
Age at first symptoms	20.21 (7.37)	18.33 (7.00)
Sex		
Male	15 (62.5)	3 (25.0)
Female	9 (37.5)	9 (75.0)
Ethnicity		
White British/Irish	21 (87.5)	10 (83.3)
Black Caribbean/African	2 (8.3)	2 (16.7)
Asian	1 (4.2)	0
Medication		
Yes	17 (70.8)	11 (91.7)
Not known	7 (29.2)	1 (8.3)
Psychotherapy		
Yes	5 (20.8)	0 (0.0)
No	8 (33.3)	5 (41.7)
Not known	11 (45.9)	7 (58.3)
Years of education	13.69 (2.72)	13.42 (2.62)
Marital status		
Single	18 (75.0)	10 (83.3)
Married or partnership	0 (0.0)	1 (8.3)
Co-habiting	6 (25.0)	1 (8.3)
Employment status		
Employed	6 (25.0)	3 (25.0)
Education/training	8 (33.3)	2 (16.7)
NEET	10 (41.7)	7 (58.3)
Previous admissions		
Yes	9 (37.5)	3 (25.0)
No	15 (62.5)	9 (75.0)

*Note:* NEET, not in education, employment, or training; PANSS, Positive and Negative Syndrome Scale.

**Table 2.** Clinical Measures at Baseline, by Randomized Group

Measure	Baseline			
	ClinTouch (*N* = 12)		Actissist (*N* = 24)	
	Mean	SD	Mean	SD
PANSS positive	17.8	5.9	16.0	3.9
PANSS negative	12.8	2.5	15.2	4.0
PANSS general	34.0	6.4	34.9	7.6
PANSS total	64.6	11.1	65.9	12.9
Calgary—mild	1.8	1.4	2.4	1.3
Calgary—moderate	1.7	1.4	1.3	1.2
Calgary—severe	2.2	1.7	1.5	1.8
Calgary—total	11.7	5.1	9.1	5.4
PSYRATS—delusions	12.5	8.8	11.9	7.3
PSYRATS—AH	13.3	15.2	16.6	14.3
PSP	51.3	13.7	48.9	11.5
GAF functioning	53.2	13.9	50.5	11.5
GAF symptoms	48.2	14.0	48.5	13.0
GAF total	46.4	14.3	44.4	10.4
PCS	19.1	8.4	20.9	8.1
ERS	77.8	8.7	82.3	7.7
EQ5D likert 0–100	58.8	21.1	64.0	16.9
Average alcohol consumption (past 30 days)	33.36 (*n* = 9 alcohol users)	45.21	23.45 (*n* = 18 alcohol users)	26.38
Number of nonsober days (past 30 days)	5.50	6.67	3.29	4.75
MARS	14.80	2.10	15.67	1.98

*Note:* PANSS, Positive and Negative Syndrome Scale; Calgary, Calgary Depression Scale for Schizophrenia; PSYRATS, Psychotic Symptoms Rating Scale; PSP, Personal and Social Performance Scale; GAF, Global Assessment of Functioning Scale; PCS, Perceived Criticism Scale; ERS, Empowerment Rating Scale; EQ5D, EuroQol-5D-5L; MARS, Medication Adherence Rating Scale.

### General Pattern of App Usage

Participants mostly interacted with the app in the latter part of the day and earlier in course of the 12-week intervention (although the target engagement rates were still achieved for the intervention period). The most popular prompted domain was voices (481 entries), followed by suspicious thoughts (404 entries), socializing (220 entries), criticism (157 entries), and cannabis (31 entries). The most popular unprompted domain was suspicious thoughts (97 entries), followed by socializing (65 entries), voices (64 entries), criticism (39 entries), and cannabis (14 entries). Considering both prompted and unprompted entries, voices overall was the most frequently accessed domain overall (545 entries), followed by suspicious thoughts (501 entries), socializing (285 entries), criticism (196 entries), and cannabis (45 entries). Expressed as an average per participants across each domain over the intervention period, participants clicked on average 1.85 times (range: 0–11) on the cannabis domain (2 outliers noted, both clicking 11 times), 1.88 times (range: 1–50) on the socializing domain (2 outliers noted, clicking 40 and 50 times), 20.88 times (range: 0–94) times on the suspicious thoughts domain (1 outlier noted, clicking 94 times), 8.17 (range: 1–25) times on the criticism domain (1 outlier noted, clicking 31 times), and 22.71 times (range: 0–174) times on the voices domain (5 outliers noted, clicking 51, 54,, 58, 74, and 174 times). These findings show that while voices was the most frequently accessed domain overall, this seemed to be influenced by a few participants who interacted with this domain quite intensively. The remaining domains appeared less influenced by outlier responses. Participants lost 2 phones during the trial. The majority of participants used a study phone (*n* = 31). There was no difference in those who used their own phone (12.5% Actissist; 8.3% ClinTouch) versus those who were loaned a study phone (83.3% Actissist; 91.7% ClinTouch).

### Secondary Outcomes

Summary statistics for all secondary outcomes across time points and conditions are shown in table 3. Inspection of the effect sizes and confidence intervals suggest that there were improvements in key outcome measures, including PANSS negative score, general and total scores, and Calgary (mild, moderate and total) scores in the Actissist group relative to the control group post-treatment. The regression coefficients (ie, adjusted mean differences) and standardized effect sizes (Cohen’s *D*) are numerically higher on these variables at the post-treatment Actissist assessment. This suggests that participants understood the content of the app and learnt new skills, improving general psychotic symptoms and mood in the short-term. Effects were not fully sustained at 22-week follow-up; although, there was no decline on any of the clinical outcomes measured.

**Table 3.** Summary Statistics and Treatment Effects at Post-Treatment

Measure	Post-Treatment				Effect (SE)	95% CI	Cohen’s *D*; 95% CI
Post-Treatment Scores	ClinTouch (*N* = 8)		Actissist (*N* = 18)				
	Mean	SD	Mean	SD			
PANSS positive	14.5	5.1	13.0	3.8	−1.30 (1.29)	−3.97, 1.37	−0.28 (−0.85, 0.29)
PANSS negative	14.0	3.9	13.3	4.5	−3.04 (1.26)	−5.64, −0.44	−0.85 (−1.58, −0.12)
PANSS general	34.5	8.7	28.4	8.8	−6.23 (2.04)	−10.45, −2.00	−0.86 (−1.44, −0.28)
PANSS total	63.0	15.6	54.7	14.6	−10.47 (3.54)	−17.80, −3.14	−0.85 (−1.44, −0.25)
Calgary—mild	2.9	1.1	1.9	1.5	−1.22 (0.58)	−2.42, −0.01	−0.92 (−1.83, −0.01)
Calgary—moderate	4.0	2.8	1.4	1.8	−2.42 (0.91)	−4.31, −0.54	−1.92 (−3.42, −0.43)
Calgary—severe	3.8	3.8	1.3	2.6	−1.92 (1.26)	−4.52, 0.68	−1.09 (−2.56, 0.39)
Calgary—total	10.8	5.1	5.1	5.1	−3.43 (1.61)	−6.76, −0.11	−0.65 (−1.28, −0.02)
PSYRATS—delusions	10.9	9.9	7.8	7.2	2.15 (3.11)	−4.30, 8.60	0.28 (−0.55, 1.1)
PSYRATS—AH	5.3	10.4	16.5	14.7	−3.07 (2.71)	−8.66, 2.54	−0.21 (−0.59, 0.17)
PSP	48.0	12.0	53.5	15.1	5.77 (4.07)	−2.64, 14.18	0.47 (−0.22, 1.16)
GAF functioning	52.8	17.7	53.8	16.3	1.02 (5.43)	−10.22, 12.26	0.08 (−0.83, 1)
GAF symptoms	54.3	16.0	57.8	15.1	3.74 (5.05)	−6.72, 14.19	0.28 (−0.5, 1.07)
GAF total	49.9	15.5	49.3	13.6	0.85 (4.87)	−9.22, 10.91	0.07 (−0.78, 0.92)
PCS	22.3	8.8	20.2	5.9	−2.13 (2.94)	−8.21, 3.96	−0.26 (−1, 0.48)
ERS	81.2	2.1	86.2	5.8	3.47 (1.95)	−0.60, 7.54	0.43 (−0.07, 0.94)
EQ5D5L likert 0–100	40.0	26.0	71.1	21.3	−117.17	−283.44, 49.10	−6.38 (−15.43, 2.67)
MARS	14.33	2.66	15.12	1.93	0.37 (0.98)	−1.67, 2.41	0.18; −0.82, 1.19
Average alcohol unit consumption over the nonsober days (last 30 days)	4.29 (*n* = 8)	3.45	8.64 (*n* = 18)	13.19	1.45 (4.10)	−7.04, 9.94	0.30; −1.45, 2.04
**22 Week Scores**	**ClinTouch (*N* = 9**)		**Actissist (*N* = 21**)		**Effect (SE**)	**95% CI**	**Cohen’s *D*; 95% CI**
	**Mean**	**SD**	**Mean**	**SD**			
PANSS positive	16.2	5.40	13.2	4.6	−1.90 (1.48)	−4.93, 1.14	−0.41 (−1.06, 0.24)
PANSS negative	13.8	4.21	13.8	4.9	−2.73 (1.33)	−5.46, 0.003	−0.76 (−1.53, 0)
PANSS general	33.6	10.14	28.9	7.5	−4.84 (2.69)	−10.37, 0.68	−0.67 (−1.43, 0.09)
PANSS total	63.6	17.54	52.9	17.4	−11.27 (6.78)	−25.19, 2.65	−0.91 (−2.04, 0.21)
Calgary—mild	2.7	1.58	2.4	1.2	−0.36 (0.56)	−1.50, 0.78	−0.27 (−1.13, 0.59)
Calgary—moderate	2.0	2.45	1.8	2.1	−0.21 (0.91)	−2.08, 1.66	−0.17 (−1.65, 1.32)
Calgary—severe	2.7	3.16	1.6	3.1	−0.44 (1.12)	−2.74, 1.86	−0.25 (−1.55, 1.06)
Calgary—total	7.3	4.82	6.0	4.9	0.57 (1.60)	−2.71, 3.85	0.11 (−0.51, 0.73)
PSYRATS—delusions	11.3	7.5	8.0	7.8	−2.11 (2.50)	−7.24, 3.02	−0.27 (−0.93, 0.39)
PSYRATS—AH	11.8	13.4	16.8	14.3	3.30 (3.95)	−4.81, 11.40	0.23 (−0.33, 0.78)
PSP	54.3	15.5	56.9	14.2	3.24 (5.49)	−8.01, 14.50	0.26 (−0.65, 1.18)
GAF functioning	57.3	10.3	59.4	14.2	3.23 (4.69)	−6.39, 12.84	0.26 (−0.52, 1.04)
GAF symptoms	49.8	17.4	55.5	16.6	5.40 (6.26)	−7.45, 18.25	0.41 (−0.56, 1.37)
GAF total	48.2	14.7	52.0	16.2	4.27 (6.27)	−8.60, 17.14	0.36 (−0.73, 1.45)
PCS	22.6	7.4	21.2	7.2	−1.54 (2.98)	−7.66, 4.58	−0.19 (−0.94, 0.56)
ERS	82.4	6.6	85.0	7.5	−0.92 (1.98)	−5.00, 3.16	−0.11 (−0.62, 0.39)
EQ5D5L likert 0–100	56.9	18.0	63.1	21.2	4.34 (6.93)	−9.89, 18.59	0.24 (−0.54, 1.01)
MARS	13.29	1.80	15.59	2.09	0.63 (0.84)	−1.12, 2.38	0.31; −0.55, 1.18
Average alcohol unit consumption over the nonsober days (last 30 days)	4.15 (*n* = 9)	3.91	4.52 (*n* = 21)	4.98	−0.14 (1.84)	−3.92, 3.64	−0.03; −0.80, 0.75

*Note:* PANSS, Positive and Negative Syndrome Scale; Calgary, Calgary Depression Scale for schizophrenia; PSYRATS, Psychotic Symptoms Rating Scale; PSP, Personal and Social Performance Scale; GAF, Global Assessment of Functioning Scale; PCS, Perceived Criticism Scale; ERS, Empowerment Rating Scale; EQ5D, EuroQol-5D-5L; MARS, Medication Adherence Rating Scale.

### Acceptability

The Actissist system was acceptable, enjoyable, beneficial, and easy to use (see supplementary table 1); 90% participants said they would recommend Actissist to others in a similar position. Illustrative feedback quotations are provided in table 4.

**Table 4.** Participant Feedback (*n* = 15)

Nature of Feedback	Illustrative Quotation
Positive views about the Actissist app	
Ease of access	“… that app what it does, it says ‘I’ve got a CPN in my pocket, I’ve got a care provider in my pocket that I can, I can go out quite freely now without my CPN I don’t have to arrange something with my CPN … It’s kind of, it gives you a bit of freedom to say ‘hold on a second, I don’t have to wait for my CPN.” (Participant 9)
	“I read the app before I went to the party, then, when I got to the party, I was in there about half an hour, twenty minutes, in, the voices I started leaving to go to the door, I wanted to get out, again, the app came into its own, I said, ‘can I just nip to the toilet quickly?’ just went to the toilet, just took out the app, just had a quick read, quick reassurance, back into the party.” (Participant 9)
	“It’s accessible, you can use it anywhere, erm in any situation, it wouldn’t be like oh you’ve got to go to the doctors or anything like that ... you can deal with it straight away.” (Participant 10)
	“If you feel, if you, if someone feeling so low and so depressed like I was …you wouldn’t want to talk to someone about those thoughts, cos they were disturbing and having that app there, just ready, like, cos it beeps, cos it beeps, whenever, every couple of hours. It was just perfect, it’s like an immediate help.” (Participant 106)
Inspires confidence and empowerment	“It’s like having somebody in the room who know’s what they’re talking about … putting confidence into you.” (Participant 132)
	“In mental health you feel a little bit like a criminal, criminalized sometimes and I think with it being on the phone it’s in your hands a little, it’s under your control a bit more, as opposed to feeling a bit like you’re under house arrest.” (Participant 11)
Facilitates self-management	“… you become your own therapist and that’s what CBT is about, being able to change your behavior … reassess a situation, about going forward on your own, uhm solution.” (Participant 5)
Becomes part of your routine	“Noticing it in an app like that, and in that order, yeah it, it’s encouraging. So you tend to get in a routine with it, which is good, or I did … and as part of your daily routine it’s like as if sommat’s looking after you, in a way, which is good.” (Participant 109)
	“… it did start to feel part of my normal routine … it was good, it was sort of like having a buddy [laughs] um so yeah every time it sort of asked you to check in it was quite a good feeling.” (Participant 7)
	“It was different, it wasn’t something I was used to, erm, and for me, it was quite good ‘cause, I kind of, I only see my care coordinator once a week, sometimes I just, I don’t like, do what she tells me but it’s like a reminder. So it kinda fits in with that for me a bit, fitted with that for me as well.” (Participant 107)
Ideas for improvement	
Minimize repetition and personalize content	“Sometimes you can get annoyed with, a bit sick of these questions, that’s all, but it’s just sometimes ’cos, ’cos you’ve heard it before, that’s all, that’s all.” (Participant 109)
Personalizing alerts to fit with lifestyle	“… it seemed like it’s prompted me too often.” (Participant 128)
	I didn’t like it when it reminded me to do it, I could do it off my own accord, when I knew I needed to do something to kill time or just to get like information out of it. Erm but, the constant reminder of it, it was just like nooo….” (Participant 111)
Depth and variety of content	“I found it was helpful at first but then I found content on the actual app was too limited… I think, there’s only so many answers, so when you answer like a question, there’s only so many like responses it can give you.” (Participant 128)

## Discussion

This is the first study to evaluate the feasibility, acceptability, and safety of a theory-driven smartphone app in a randomized controlled design compared against an active control condition in early psychosis. Benefits reported for psychosis outcomes were observed in comparison to a similar smartphone app platform for tracking mental health in psychosis. Specifically, we showed that Actissist was feasible, acceptable, and safe, with high levels of satisfaction and indications of a beneficial effect, even over an active control group (symptom monitoring). Retention in the study was excellent (35/36 participants; 97%), which reflects other DHI retention rates of around 92% psychosis patients remaining in the trial until the end.^11^ Assessment completion was high and reflects similar DHI trials in psychosis. Engagement with the system was high (75% participants used the app at least once/day), which reflects engagement with other alert-based DHIs in psychosis (average response rate to alerts across trials of 71.9%, with 86.5%–94% participants interacting with the app on the predefined study days).^11^ Feedback from participants was overwhelmingly positive, suggesting that participants enjoyed using the app, understood the content of the app, with some suggestion that participants implemented new skills in the course of their day-to-day lives, evidenced by the promising treatment effect estimates. Findings sit alongside those emerging in the psychosis literature, which shows that digital symptom monitoring systems are feasible and acceptable to patients.^2,22,23,43^

The treatment effects immediately post-treatment favoured the Actissist group, and were large. This suggests that the Actissist app conferred added benefit over and above routine symptom monitoring in terms of negative, general and total psychotic symptom scores and mood in the short term. Although there was no decline on any of the clinical outcomes measured at 22-week follow-up, treatment estimates were not maintained at this time-point, suggesting that further testing of sustained effects over time is needed. Furthermore, participants tended to use the app later in the day, reflecting the importance of using personalized alerts rather than pseudo-random alerts over a prespecified time period within days.

Further research should be hastened in light of digital health care initiatives that lack an evidence base, and future consideration evaluating fast-paced technological innovations outside an RCT context is needed. There is a need to ensure parity and to limit the exclusion of low-income individuals who cannot afford smartphones and their associated cost. This study has notable strengths. We used an active control symptom-monitoring app condition, which matched the overall look-and-feel and functionality of the Actissist app, thus accounting for the nonspecifics of smartphone use. The apps were different, however, in that ClinTouch is designed to be completed in-the-moment and cannot be left to complete at a chosen time, whereas Actissist acts as more of an active and responsive self-management tool and is therefore available whenever the user requires (either prompted or self-initiated). We implemented a rigorous reporting SAE procedure for both groups. Trials without an active control condition have problems gathering a true measure of SAEs as researchers typically have less contact with the TAU group than the treatment arm. Finally, raters were blind to group allocation and stakeholders were co-designers of the system. There are some limitations. Participants were incentivized to use both apps, which may have increased usage beyond what would be observed in a real-world setting. Second, we operationalized engagement according to experience sampling methodology criteria (completion of 33% data entries over the intervention period). As this study was incentivized, we needed to apply a criterion to prevent participants from artificially inflating app usage to obtain incentives. In future DHIs, we suggest researchers report app usage more descriptively rather than prespecify completion rates.^44,45^ Finally, whilst the small sample could impact generalizability of findings, participants were representative of a help-seeking early psychosis group.

Our findings suggest that the Actissist system may confer additional benefits over routine mobile symptom monitoring in the short term. Participants were engaged, active, and adherent with the system; therefore, findings justify proceeding to a fully powered trial. This study represents an important and significant step toward developing a technology platform for delivering a range of psychosocial interventions for psychosis. Indeed, this study is the first to show that an active self-management app can potentially improve outcomes in psychosis, even beyond a passive symptom-monitoring app. It shows how proof-of-concept trials can underpin digital experimental health care with empirically derived theoretical frameworks. If trials such as Actissist are effective, a major challenge is for mental health services to recognize and incorporate DHIs into the health care setting.

## Supplementary Material

Supplementary data are available at *Schizophrenia Bulletin* online.

Supplementary Table 1

## Funding

The work was supported by the Medical Research Council Developmental Pathway Funding Scheme (grant number MR/L005301/1) and was supported by the University of Manchester and Greater Manchester Mental Health NHS Foundation Trust.
