# Corrigendum

**DOI:** 10.1093/schbul/sby055

**Published:** 2018-04-25

**Corrigendum to “Actissist: Proof-of-Concept Trial of a Theory-Driven Digital Intervention for Psychosis”** by Sandra Bucci et al. Schizophrenia Bulletin, 2018. doi: 10.1093/schbul/sby032.

The Conflict of Interest and Funding sections were erroneously omitted from the article. They have been added.

The author regrets the error.

