# Supplementary material for: Actissist: Proof-of-Concept Trial of a Theory-Driven Digital Intervention for Psychosis
Source: Schizophr Bull. 2018 Mar 16;44(5):1070–80. doi: 10.1093/schbul/sby032 (PMC6135229; doi:10.1093/schbul/sby032)
Supplement: Supplementary Table 1 [file sby032_suppl_supplementary_table1.docx]

**Supplementary Table 1: Quantitative feedback scores for Actissist app (post-treatment; n=24)**

| **Item** | **Mean (SD)** | **Min** | **Max** |
| --- | --- | --- | --- |
| Did answering the questions take a lot of work? | 2.85 (1.80) | 1 | 7 |
| Were there times when you felt like not answering? | 4.92 (2.06) | 1 | 7 |
| Did answering the questions take up a lot of time? | 2.38 (1.33) | 1 | 6 |
| Were there times when you had to stop doing something in order to answer questions? | 4.42 (1.90) | 1 | 7 |
| Was it difficult to keep track of what the questions were asking? | 2.77 (1.75) | 1 | 6 |
| Were you familiar with using this type of technology? | 5.46 (1.96) | 1 | 7 |
| Was it difficult to keep track of what the questions were asking? | 2.92 (1.81) | 1 | 6 |
| Did you ever lose or forget the device? | 3.28 (1.93) | 1 | 7 |
| Was using the touchscreen difficult? | 1.50 (1.14) | 1 | 6 |
| Do you think other people would find the software easy to use? | 5.96 (1.43) | 1 | 7 |
| Do you think that you could make use of this approach in your everyday life? | 4.96 (1.71) | 1 | 7 |
| Do you think that this approach could help you or other service users? | 5.58 (1.33) | 3 | 7 |
| Overall, this experience was stressful | 2.12 (1.45) | 1 | 5 |
| Overall, this experience was challenging | 2.38 (1.50) | 1 | 7 |
| Overall this experience was pleasing | 4.42 (1.68) | 1 | 7 |
| Did filling in the questions make you feel worse? | 2.15 (1.67) | 1 | 6 |
| Did filling in the questions make you feel better? | 4.31 (1.81) | 1 | 7 |
| Did you find the questions intrusive? | 2.54 (1.90) | 1 | 7 |
| Was filling in the questions inconvenient? | 3.31 (2.11) | 1 | 7 |
| Did you enjoy filling in the questions? | 3.96 (1.59) | 2 | 7 |
| Total QFQ score | 71.24 (10.15) | 56.00 | 91.00 |
